# Radicalizing Hope

**DOI:** 10.1007/s11673-023-10291-2

**Published:** 2023-09-07

**Authors:** Michael Chapman, Paul Komesaroff

**Affiliations:** 1 Department of Palliative Care, Canberra Health Services, Garran ACT, Canberra, Australia; 2 Medical School, College of Health and Medicine, Australian National University, Canberra, Australia; 3 Monash University, Clayton, Victoria Australia

**Keywords:** COVID-19, Hope, Courage, New-normal, Bioethics, Global health

## Abstract

The race against COVID-19 has been intense and painful and many of us are now looking for a way to move on. We may try to seize a degree of comfort and security by convincing ourselves that we are among the “fittest”—that is, among those who have managed to survive—who can now hope for a “new-normal” time, relatively unscathed. But this isn’t what we should be hoping for. Our world, and ourselves, will never be free of COVID-19 or its insidious effects. COVID-19, like climate change, is a threat multiplier and the challenges it has raised are now indelibly engraved in our vulnerable, interconnected lives. Rather than vainly hoping for a return to an imaginary, erstwhile “normal” what we need is something more fundamental: a new version of hope that embraces a courage to learn what we need to do, to enable us to live a future to which we aspire. Perhaps counter-intuitively, we need to accept that the COVID-19 experience has already changed us deeply and hope that we can learn from this and from the future changes that the pandemic will give rise to. We need to radicalize our responses to the challenges, enabling ourselves to learn new lessons about old but increasingly pertinent topics, such as the realities of human fragility, and inter-connection.

While one of us (MC) was waiting outside his son’s school he had a chance conversation with another parent on the continuing presence of COVID-19 in aged care facilities. At that stage case numbers were increasing in Australia and more people were dying than ever before. The parent’s response was, “It’s survival of the fittest, I guess.” Putting aside the crude misstatement of the Darwinian idea of evolutionary “fitness,” this response to a major crisis that has caused immense suffering to so many people says a lot about current social attitudes, not just to the pandemic but also to what might once have been regarded as an irrefragable responsibility to protect the frail and vulnerable in our midst. It implies that our fellow-parent considers it ethically permissible for us to ignore the needs of citizens who are elderly, sick, or otherwise in need, and that it is somehow permissible merely to allow them to die. Worse still, it suggests that the vast effort that has gone into resisting and responding to COVID-19 has given way to an indifference to its tragic outcomes and a quietistic sense that we’ve done enough to mitigate its impact, so that any deaths that now occur are to be expected and endured.

Maybe this is reading too much into what was undoubtedly intended as a throwaway line. However, intriguingly, our fellow-parent’s statement seems to strike at something that has been repeatedly hinted at during this pandemic. It suggests that there is a growing sense that it is acceptable to calculate the value of people according to their physical—or maybe cultural—characteristics. Such estimations and conflations of value and vulnerability seem to have increasingly served as a basis for determining access to healthcare or other social resources during the pandemic. Further, occurring in the course of a conversation between relatively young and healthy people, the comment implies that the ills of the pandemic are tolerable as long as they only affect those who aren’t like me, the “fit” in this case. However, as some of us realize, separating those who are vulnerable to the impact of COVID-19 from those that aren’t is more of a challenge than it seems. While the risk of severe illness and death is higher for those whose health is obviously fragile, who are older and who have more chronic diseases, the situation is clearly more complex. For example, those most “fit” (in our fellow-parent’s sense) are likely to be those with a combination of invisible immunological factors, who may turn out, perversely, not to include ourselves (Nagarkatti and Nagarkatti, 2020). The uncertainties associated with the risk of a prolonged impact of “long COVID” following an acute infection, not to mention the acknowledged vulnerability of individuals from certain economic or cultural backgrounds, shows that increased risks associated with COVID-19 are not just a matter of apparent robustness or its absence. COVID-19, like climate change, is a “threat multiplier” which compounds other risks, challenges, and vulnerabilities to which individuals may be exposed (Sydney 2021). While many of us have already lived through episodes of COVID-19 infection very few can claim to have been left completely unscathed by it.

Perhaps, however, it is more useful to think of our fellow-parent’s statement as expressing a particular, slightly desperate, concept of “hope.” They are hoping, maybe, that their survival is assured because the COVID-19 experience is about to end. From this perspective the pandemic is something that they nervously have to wait out. This idea echoes the metaphorical idea that the response to COVID-19 is a “race” that humanity needs to win. Governments, corporations, nations are frantically striving to develop, test, and roll-out vaccines and treatments before it is too late (Boseley 2021). Here, while admitting the urgency, however, pursuing the metaphor, many have cautioned against viewing COVID-19 as a “sprint” but have rather advocated that our struggle be viewed as a kind of marathon (Kousta 2021). Nonetheless, regardless of speed or duration, there is a concealed confidence that sooner or later the race will conclude and the competitors will return home. For the fit this implies a need to endure until the end, when they will be rewarded with the prize of a “new normal” experience of life after COVID-19.

Other kinds of hope are likely to be active here too. One relates to a belief that the weapons we have, or will have, will ultimately defeat the COVID-19 enemy. While early in the pandemic an emphasis had been placed on the power of community cooperation and collaborations as defence against the pandemic, the primary hope for a definitive solution quickly focused on the development of new technologies. Whether this emphasized the capacity of IT solutions to connect us despite necessary physical distances and digital surveillance to limit COVID-19 through monitoring and controlling behaviour or the belief that medical and vaccine technologies would produce cures for or protections against all that COVID-19 could throw at us, the central core of this version of hope was a faith in the ability of science and technology to overcome all imaginable obstacles.

There is now considerable evidence that some aspects of these hopes have been well founded while others are over-stated. Millions of people have died and countless others have experienced profound changes in the routines and structures of their lives, but for many, much of what is regarded as normal experience has been restored. In many countries public health restrictions and PPE requirements have been wound back. Technologies that were once seen as foreign and threatening, such as CCTV surveillance, video conferencing, and novel treatments and vaccinations, have become commonplace. These outcomes have become established even in the face of vigorous, sometimes violent, disputes involving some community members around public health advice, social restrictions, vaccination mandates and other issues, often linked to discomfort about the blurring of boundaries between personal life-worlds and the public sphere of political and ethical discourses (Albrecht 2022).

The varied implications of the hopes that have been achieved through the pandemic seems a salient reminder that our hopes aren’t always ethically sound. Be it the more obviously problematic hope of our fellow-fit-parent that they, as compared with everyone, escape COVID-19, or the more subtle complexity of the potentiation of inequity and inequality that can be associated with hope that technology alone can solve global, human problems, our hopes have real moral content. They reveal something about how we understand and engage with the world we feel we live in and the world we aspire to create for ourselves. Now that some time has passed and experience has been gathered, we are in a position to assess whether the hopes on which we had come to depend were the best and most appropriate ones to adhere to. While challenging, undertaking such an analysis may allow us to entertain novel questions about how we had imagined the crisis and about the future we now expect to unfold. It may even force us to think about how our hopes relate to our responses to other global challenges and tragedies. As with COVID-19, possible future catastrophes may threaten not only the stability of the systems of meaning on which we depend but even the structures of thought on which we rely to construct them. Based on what we now know, to what hopes should we turn?

Widely shared hopes of surviving and overcoming challenges may provide some semblance of solace in times of crisis but they are not always helpful. Hopes which are just superficial optimism, self-focused or mired in ignorance or false beliefs may not be ethically sound at all. For hopes to be ethically sound they likely need additional qualities like being wise and courageous. But, sometimes even this may not be enough. Sometimes, more radical hopes are required. In his book exploring this notion, the anthropologist and psychoanalyst Jonathan Lear tells the story of Plenty Coups, a famous chief of the Crow nation, the members of which once roamed the northwest plains of the United States (Lear, 2008). Plenty Coups had a series of dreams as a young man which he and his tribe interpreted as a warning that the white man would threaten the life of the Crow people and that this would change everything. Plenty Coups’ response, as Lear interprets it, was to be able to maintain both “hope” and “courage” (ideas drawn respectively from Kant and Aristotle) through a drastic reimagining of what those words could mean. Like others facing the dissolution of ordinary certainties, whether as a result of social change, as in this case, or following intense personal trauma, new cultural, psychological, and ethical pathways to the future had to be constructed.

The pre-existing martial concept of courage for the Crow didn’t fit the new world that the white man would bring where everything was subject to change. Plenty Coups therefore sought to emulate the chickadee—a bird that gains its knowledge from others—and learn new ways of responding, of acting courageously and of constructing a future world of meaning. While these new ways were completely out of step with traditional tribal beliefs they were able, in Lear’s interpretation, to prepare the Crow for adapting to their new circumstances more successfully than many of the other tribes of the time. In other words, courage, for Plenty Coups, had to be disengaged from the values and meanings previously associated with it that no longer applied. Instead, a different architecture was needed which allowed Plenty Coups and his people to rethink basic categories of temporality, subjectivity, and meaning. Together, they revised much of what they had previously taken for granted and replaced it with a system of concepts that allowed them to project themselves towards a new set of ethical values that they could not even yet articulate. They did not assume that survival would result merely from their remaining “fit” versions of the old style of Crow people: they needed to become something new. But, they did this together. Radically re-imagining structures of identity and value was a shared process that reinforced their commitment to each other as a people. In fact, it was their search for what it meant to be a Crow person in this new time that enabled them to remain strong and connected.

Plenty Coups was guided by a vision to realize that his world had changed and that a passive faith that his people could survive without changing themselves was unlikely to be realized. Arguably, the same fundamental insight applies today. As a global community, we have largely ignored the lessons of previous pandemics, which bear a close similarity to those of COVID-19 (Smith and Upshur 2020). A key effect of the widespread social and economic disruption has been a rapid exacerbation of the suffering of poor and disadvantaged groups. Catastrophic events, such as the war in Ukraine—which has drawn vivid attention to the possibility at any moment of a global nuclear holocaust—show how little has been learnt from the tragedies of the great European wars of the twentieth century. One of many unrecoverable costs of the pandemic is the lost years of failure to respond to the intensifying environmental crisis, which is the sordid legacy of centuries of economic expansion and political neglect (led, of course, by the “fittest” and least vulnerable nations and people) (Loschke 2022). While COVID-19 may have briefly interrupted some aspects of the onslaught on our planet, the focus on fossil fuels to support economic recovery, and the refusal even to consider issues like those of the micro-plastic pollution exacerbated by the mountains of PPE produced in response to it, highlights vividly the self-destructive blindness that is likely propelling us towards an unprecedented global disaster (Chien et al. 2021; Haque and Fan 2022).

If we are to be able to move in a different direction we will have to radicalize our hopes. We will have to repudiate some conventional ways of living and direct ourselves towards fresh goals that respond to the challenges we now face. “We” will have to do this, because, like the Crow people, we’re all in this together, continuously connected through our lives, lived in response to others, and our world as the determinative source of who we are and what we do. While a fixed sense of a “new normal” might provide a brief illusion of comfort, it is rather towards new possibilities and hoped for futures that we need to direct ourselves. Central to this, for instance, could be the radical hope that we do not “survive” COVID-19 unscathed, but that we welcome the fact that our lives have been fundamentally changed. Arundhati Roy has written wryly that “nothing could be worse than a return to normality,” hoping that the pandemic could act as a “portal,” a juncture between our past ideas and habits and more useful, fair and sustainable ones of the future (Roy 2022). Many such radical hopes have the potential to realize this kind of changed world arising from COVID-19. Of these, two seem absolutely unavoidable: that we learn to live within vulnerability and that we recognize our inseparable, interdependence with others and our world around us.

While those whom our fellow-parent presumably considers to be the “fittest” may have a greater chance of surviving a COVID-19 infection in the short term, the mounting risk of additional illnesses, injuries, and harms, including the threat of other zoonotic infections, bereavements, and loss of economic security, provides a dire reminder that vulnerability is a common human experience. While we place great value on independence and economic productivity, our most poignant and fecund experiences are often those characterized by dependence and frailty, as for instance in infancy and in ageing (Nussbaum 2004). Radicalizing hopes in response to COVID-19 might include some turning away from the notion that there will ever be a technological cure for our vulnerability. Instead, perhaps a key learning from our responses to COVID-19 is that our capacity to work for the general benefit of the others around us is profound when we appreciate the shared need to do so. Lear argues that courage is virtuous because it provides an effective way of coping with the challenges posed by our very mortal and worldly existence. It offers ways of living well within the limits that we all face, regardless of whether we can discern them distinctly.

Whether it is courageous to have confidence in one’s ability to overcome life’s risks is arguable. Many of the challenges and outcomes are unpredictable but our living vulnerability is a certainty. We have been slow to acknowledge that this is a condition that is not unique to humankind. The natural environment is exquisitely vulnerable, as has been vividly demonstrated already to our great cost. The recognition of this vulnerability involves accepting that being “fit” is largely a matter of context, time, and no small amount of luck. Radical hope in response to vulnerability does not imply fatalism: rather, it opens awareness to the fact that while life is fragile and change unavoidable, how life is valued and celebrated is a matter of conscious and determined choice. As the Australian climate scientist Joëlle Gergis noted this is true of us as individuals and as members of a biosphere facing a climate crisis, our “scars (can be) a celebrated symbol of resilience, of life holding on” (Gergis, 2022). Through embracing an awareness that we will decline, and that we will die, we may be able to live with more joy, more compassion, and less paralysing fear of these unwanted but inevitable realities.

Even though vulnerability is universal, how it is encountered by an individual is infinitely variable. Many have noted that COVID-19 has had a disproportionate impact on the most vulnerable nations and people of our world. This has led to intensified social, financial, and health inequities and disadvantage (Stroud 2020). Whether one considers oneself a member of these communities it is necessary to focus on redressing this injustice. Our connection to each other and to our world could not be more clearly displayed than it has been in the last few years. COVID-19 entered the global community through a market in Wuhan and then in a matter of weeks spread around the world, laying bare with vivid clarity the intricacy of our entanglement within the biosphere.

Our lives touch and then change each other. We cannot separate ourselves from others. Even during the lockdowns imposed to secure the health of communities, it was only through the preservation of interconnections that society was able to continue functioning. Even when physical distancing drew people apart spatially, the multiple dimensions of social interconnections and mutual reliance endured unimpeded (Sorensen, et al. 2021). Despite contemporary ideologies of freedom and individual autonomy, this mutual reliance is irreducible. During the pandemic a focus on the efforts of (usually well reimbursed) frontline healthcare workers often overlooked the roles of much less well supported workers in other critical industries, like transportation and the gig economy. In the face of global and universal needs, opportunities to rise above structural inequities within healthcare practices have often been missed. With increasing pressures on global resources, particularly given the likelihood of further social and economic disruption due to climate change, the global community will increasingly rely on an ability to recognize interconnection and interdependence.

Although our fellow-parent may still not realize it, the race against COVID-19 is far from being won. If anything, the lesson we have yet to learn is that we remain at continuing risk of falling further behind unless we continue to pick up the pace. This will require a radicalization of hopes for a world after COVID-19 and a rejection of the idea that a return to “normal” is sufficient or acceptable. The lessons are not new because the hope that shared vulnerability and interconnection might be recognized has endured for thousands of years. Our experiences remain intertwined, increasingly so within the inter-connections of the contemporary world. Given the opportunities we’ve had up until now, perhaps it is radical to insist we have the capacity to learn these lessons, but the need has never been more urgent.
